# Cell Cycle-Related Cyclin B1 Quantification

**DOI:** 10.1371/journal.pone.0007064

**Published:** 2009-09-18

**Authors:** Phyllis S. Frisa, James W. Jacobberger

**Affiliations:** Case Comprehensive Cancer Center, Case Western Reserve University, Cleveland, Ohio, United States of America; Roswell Park Cancer Institute, United States of America

## Abstract

**Background:**

To obtain non-relative measures of cell proteins, purified preparations of the same proteins are used as standards in Western blots. We have previously quantified SV40 large T antigen expressed over a several fold range in different cell lines and correlated the average number of molecules to average fluorescence obtained by cytometry and determined cell cycle phase related expression by calculation from multi-parametric cytometry data. Using a modified approach, we report quantification of endogenous cyclin B1 and generation of the cell cycle time related expression profile.

**Methodology:**

Recombinant cyclin B1 was purified from a baculovirus lysate using an antibody affinity column and concentrated. We created fixed cell preparations from nocodazole-treated (high cyclin B1) and serum starved (low cyclin B1) PC3 cells that were either lyophilized (for preservation) or solubilized. The lysates and purified cyclin B1 were subjected to Western blotting; the cell preparations were subjected to cytometry, and fluorescence was correlated to molecules. Three untreated cell lines (K562, HeLa, and RKO) were prepared for cytometry without lyophilization and also prepared for Western blotting. These were quantified by Western blotting and by cytometry using the standard cell preparations.

**Results:**

The standard cell preparations had 1.5×10^5^ to 2.5×10^6^ molecules of cyclin B1 per cell on average (i.e., 16-fold range). The average coefficient of variation was 24%. Fluorescence varied 12-fold. The relationship between molecules/cell (Western blot) and immunofluorescence (cytometry) was linear (r^2^ = 0.87). Average cyclin B1 levels for the three untreated cell lines determined by Western blotting and cytometry agreed within a factor of 2. The non-linear rise in cyclin B1 in S phase was quantified from correlated plots of cyclin B1 and DNA content. The peak levels achieved in G2 were similar despite differences in lineage, growth conditions, and rates of increase through the cell cycle (range: 1.6–2.2×10^6^ molecules per cell).

**Conclusions:**

Net cyclin B1 expression begins in G1 in human somatic cells lines; increases non-linearly with variation in rates of accumulation, but peaks at similar peak values in different cell lines growing under different conditions. This suggests tight quantitative end point control.

## Introduction

To a very large extent, our knowledge of cellular regulation at the molecular level rests on simple biochemical reactions correlated with genetic interactions and “molecular genetic” interventions, which are related to phenotype. In general this knowledge is communicated with cartoons coupled with somewhat tortured text to create a sense of understanding. Most of this knowledge is not quantitative and the limited quantitative knowledge is based on relative measurements. In recent years, momentum and enthusiasm has grown for more integrated systems approaches to the study of cell biology [1]. One cornerstone of these “Systems Biology” efforts is mathematical modeling of the regulatory biochemical network of the cell [2]. Our short term purpose for the work reported here is to transform cytometry data into non-relative measurements. The long term goal is to create a robust system of measurement on an absolute scale that supplies the information that these systems efforts require.

Quantification of specific cell protein contents is generally measured and reported as relative values. There are exceptions and these usually involve incorporating purified proteins as standards in immunoblotting methods [e.g., 3], measuring fluorescence of fluorescent proteins (FP) or of FP-fusion proteins and external fluorescent protein standards on the same instrument [4], and mass spectrometry [5]. Only the FP method provides single cell resolution. We have previously coupled the immunoblot approach with cell line standards, expressing different levels of SV40 large T antigen (Tag) expressed over a several fold range, to create a method whereby protein expression could be quantified in absolute terms (molecules per cell) and correlated with any other parameter by multi-parametric cytometry of the same cell lines [6]. The method relied on correlating the average Tag-specific fluorescence with the Optical Density (OD) of the Tag band on western blots into which recombinant, purified SV40 Tag was also incorporated. Because cytometry was used, this was also a single cell method.

Here we report that we have extended this procedure to a protein that oscillates during the cell cycle, cyclin B1. SV40 T antigen is a long-lived protein expressed throughout the cell cycle when expressed from its own promoter [7] or from recombinant promoters from other viruses [8], [9]. Overall, Tag is in equilibrium at an expression rate that ranges less than two fold as a ratio of the average G2 to average G1 cell. On the other hand, cyclin B1 ranges from an undetectable level in early G1 to 1000's fold over the earliest detectable levels with a very sharp, non-linear rise in late S and G2 [10]. We faced several challenges for cyclin B1. First, we did not have an obvious strategy to create cell lines that expressed the protein over the many-fold range necessary to create a standard curve. Second, the antibodies available to detect it had significantly lower affinity than the Tag antibody; third, related to the first, cyclin B1 was thought to be expressed at low levels compared to a stable, promiscuously expressed protein like Tag. Finally, we did not know whether the fluorescence averaging method would work well when a large number of the cells were not expressing the protein. In principle, this shouldn't matter, but coupled with the relatively low expression of cyclin B1, a significant fraction of the fluorescence signal is background. The last three problems are all related and depend on the quality of the antibody.

Other investigators have measured cyclin B1 levels in HeLa cells [4], [11], mouse and Xenopus oocytes [12], [13], and yeast [14]. The methods and results vary significantly. Here, we report results for Hela, RKO, and K562. The methods used to quantify were substantially different in each case and the direct comparisons of Hela cells are not in agreement. The results of Xu et al. agree with our results within two-fold. The results of Arooz et al. are different by a large factor. It is not clear whether this is technical or biological, although the difference in magnitude seems too great to be biological, and examination of methodological points wherein differences might occur on the order of two-fold doesn't account for the orders of magnitude difference in results.

## Results and Discussion

### Effect of Protein Concentration on Immunoblot Detection of Purified Cyclin B1

Our previous experience with SV40 large T antigen (Tag) showed that there were two important facets to Western blotting that should be addressed for molecular quantification [6].

**Figure 1:**
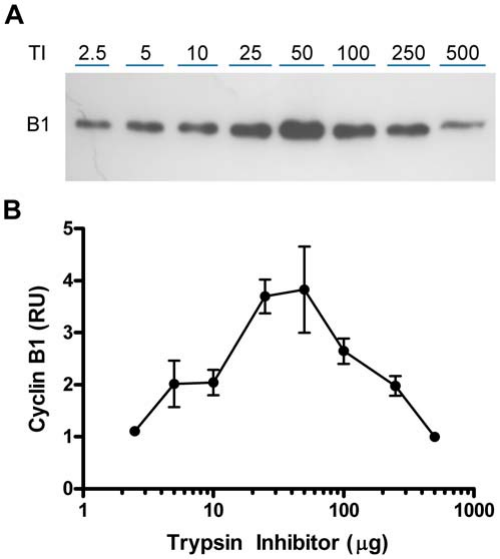
Protein level affects epitope detection. (A) A fixed amount of purified cyclin B1 (6.36 ng) was added to a serial dilution of trypsin inhibitor (TI) and subjected to electrophoresis and Western blotting. (B) Relative intensity of the cyclin B1 bands is presented corresponding to trypsin inhibitor levels. Cyclin B1 intensity was maximal at 25–50 µg trypsin inhibitor and decreased at lower and higher protein levels. Densitometry readings from 3 experiments and 6 gels were combined by normalizing by linear regression on measurements common to each paired series. The number of common points ranged from 6 to 8. The combined set was then normalized to the 500 TI mean value, therefore the Y axis can be read as fold over this value.

The quantity of protein loaded becomes important because the purified recombinant proteins need to be applied as a dilution series to create the standard. Protein content per sample affects the distance a protein runs and the intensity of the band transferred to the paper. Previously, we used NIH 3T3 cell lysates to equalize the total protein levels in the recombinant dilutions of Tag. For cyclin B1, we needed a non-replicating cell source or a replicating source from a non-cross reacting species. Since either of these would involve some difficulty, we investigated use of a single, purified protein in lieu of cell lysates. We tried three different proteins. A typical Western blot for trypsin inhibitor is shown in Figure 1A. In this experiment, a constant amount of recombinant cyclin B1 was mixed with variable amounts of trypsin inhibitor, which, at 21.5 kDa, migrates faster than cyclin B1 during electrophoresis. The intensity of the cyclin B1 band changed in the expected manner as a function of trypsin inhibitor concentration. The results of 3 experiments were combined in Figure 1B and show that 25–50 µg of trypsin inhibitor provide an optimal cyclin B1 signal. Beta-galactosidase, which migrates slower than cyclin B1, showed the same pattern of increase in cyclin B1 intensity as trypsin inhibitor. BSA, which migrates at about the level of cyclin B1, interfered with cyclin B1 migration. In subsequent work, trypsin inhibitor was added to the recombinant cyclin B1 and the cells lysates to bring the total protein in each electrophoretic lane to 50 µg/lane. We conclude that adding a purified protein is an efficient way to optimize for protein concentration effect, however, the type and amount appears empirical. For example, similar experiments for Tag showed peak detection at 1.5 µg added β-galactosidase.

**Figure 2:**
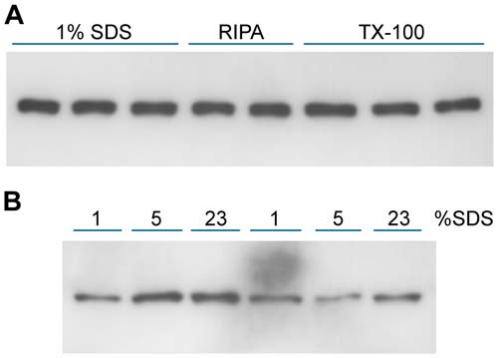
Extraction of cyclin B1 in various lysis buffers. Equal numbers of dissociated PC3 cells were solubilized in lysis buffer containing RIPA, Triton X-100 or SDS (see Materials and Methods) and analyzed for cyclin B1 by Western blot (A). Increasing the SDS level to 23% did not release more cyclin B1 (B). There was some variability between lanes but, based on multiple gels, it was clear that cyclin B1 was readily extracted by all methods.

The method of cell lysis is the second major variable. The validity of quantitative Western blotting is dependent on solubilization of all or most of the protein under study. This was difficult for Tag because a significant fraction of Tag is tightly bound to the nuclear matrix. To test whether cyclin B1 posed similar problems, lysis buffers of different strengths were used to extract cyclin B1 from PC3 and DU145 prostate cell lines. RIPA, 0.75% Triton X-100, and 1, 5, and 23% SDS did not show major reproducible differences in cyclin B1 solubilization (Figure 2) indicating that it is not tightly bound to insoluble cellular structures. This was verified in additional experiments in which cyclin B1 was not detectable by immunofluorescence cytometry or Western blot of cells after Triton X-100 permeabilization and washing (data not shown). SDS at 1% was used in subsequent work.

### Manipulation of Cyclin B1 Levels in Cell Lines

**Figure 3:**
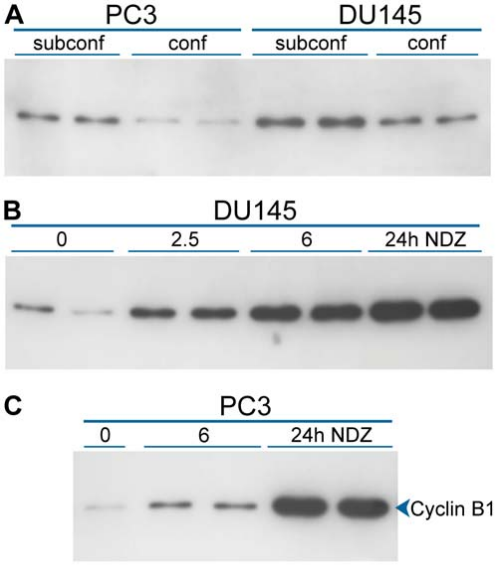
Manipulation of cyclin B1 levels. Lysates were made from known numbers of PC3 and DU145 cells that had either been grown to confluence or harvested at very low density. Equal numbers of cells were loaded in each lane and Western analysis of cyclin B1 was performed. The increase in total cyclin B1 in subconfluent relative to confluent cells was 4.4 fold for PC3 cells and 2.5 fold for DU145 cells (A). Nocodazole (NDZ) treatment for the indicated hours produced a final 17.6 fold increase over confluent cells for DU145 cells (B) and a 52 fold increase for PC3 cells (C).

Our perception was that relative average levels of cyclin B1 do not vary over a wide range between human cell lines or stimulated peripheral blood lymphocytes. Therefore, to create cell preparations with a wider range, we manipulated the average expression of cyclin B1 for cell populations by altering growth conditions. Cyclin B1 is expressed at levels at or below the level of detection in early-mid G1 [15], [16] and is thought to be essentially absent in G0, therefore, growing cells to confluency or serum starvation should decrease the average expression of cyclin B1. Conversely, growing cells at a low density (3000–5000 cells/cm^2^) enhances growth rate and therefore increases the number of cells in G2 and M, during which cyclin B1 levels peak. These extremes resulted in 3 and 4 fold ranges in average cyclin B1 expression for DU145 and PC3, respectively (Figure 3A). To obtain populations with higher average levels of cyclin B1, DU145 and PC3 cells were treated with nocodazole to arrest cells in metaphase. Cyclin B1 is normally at or near peak levels in metaphase. Additionally, when cells are synchronized by any of several methods [17], [18] or arrested with nocodazole (this paper), cyclin B1 levels per cell increase to supra-normal levels as a function of time. Compared to confluent cultures, cyclin B1 intensity of treated DU145 cells increased 18 fold at 24 h (Figure 3B). Nocodazole treatment of PC3 cells increased cyclin B1 levels 7 fold at 6h and 52 fold at 24 h (Figure 3C).

### Cyclin B1 Standard Cell Preparations

For standard cell preparations, nocodazole treated K562 cells that normally express higher levels of cyclin B1, established the high end of a standard set, and untreated 22Rv1 that normally express lower levels defined the low end. For intermediate levels, we used 2 batches of PC3 cells that were either confluent or had been treated with nocodazole and mixed them in different proportions. This provided fixed-cell preparations for cytometry and lysates for Western blotting that evenly spanned a wide range of average cyclin B1 levels.

**Figure 4:**
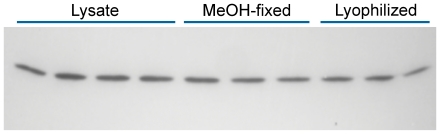
Comparison of MeOH fixed cells to MeOH fixed-lyophilized cells. A culture of PC3 cells was trypsinized, washed, and then divided into three equal parts. One part was solubilized for electrophoresis, one part was fixed with methanol, and the third part was fixed with methanol and then lyophilized. The methanol-fixed and lyophilized cell preparations were washed then solubilized with 23% SDS. The lanes were loaded with equal numbers of cells.

For cytometry, we fixed large amounts of the standard samples to be used for future experiments. To enhance long term storage of these samples, we lyophilized the cytometry samples in multiple vials and stored them at −80°C. Figure 4 shows that lysates of lyophilized, MeOH-fixed, and freshly prepared PC3 cells do not demonstrate significant differences in cyclin B1 levels. MeOH-fixed cells, that are normally stored in 90% MeOH, lose immuno-reactivity as a function of time are not quantitatively useful after long periods (6 months – 1 year; unpublished data).

### Cyclin B1 Quantification for Standards by Western blot

**Figure 5:**
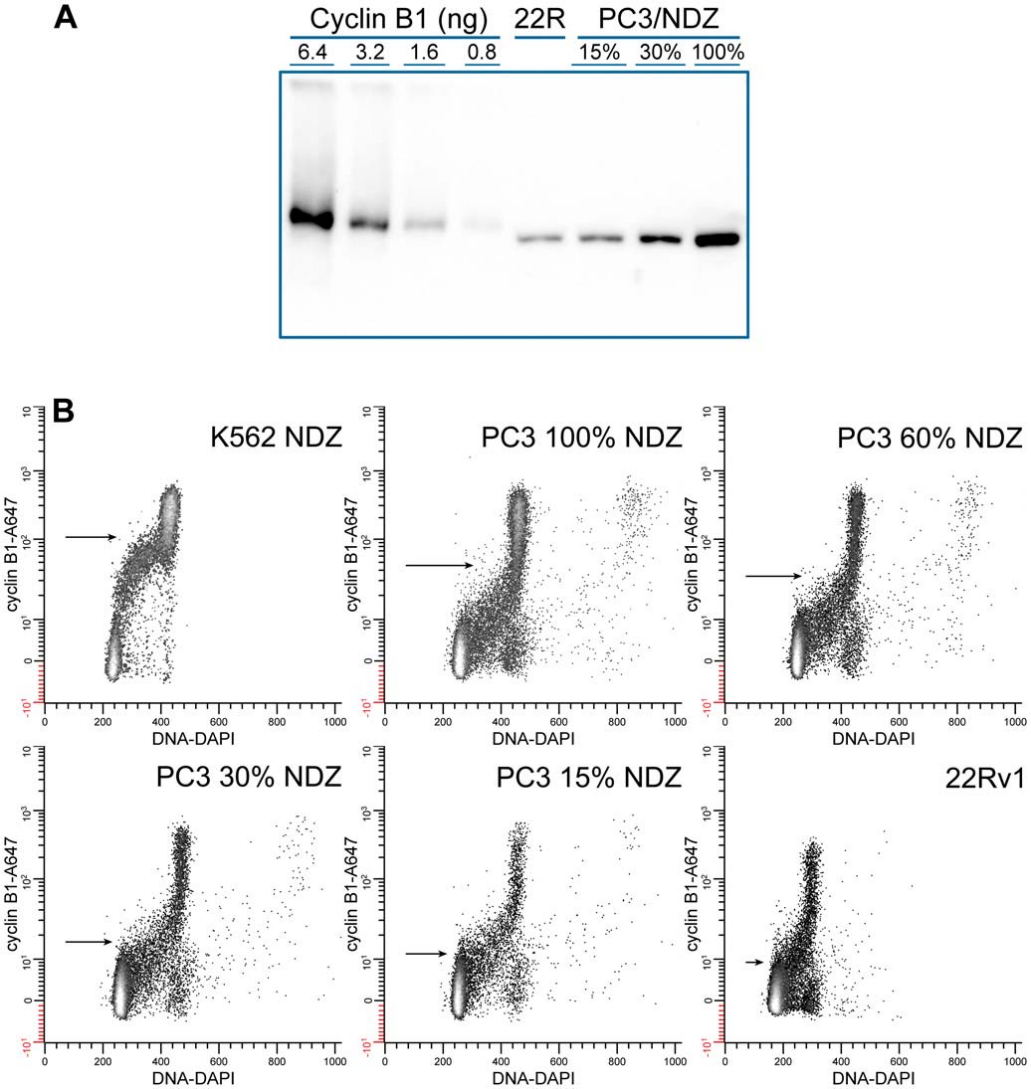
Cytometry and quantitative Western blots of standards. (A) Left 4 lanes are recombinant cyclin B1 at two-fold dilutions. Recombinant cyclin B1 and lysate concentrations were adjusted so that the band intensities for recombinant cyclin B1 spanned the range of those of the lysates. Trypsin inhibitor was added to each sample to bring the total protein per lane to 50 µg. (B) Lyophilized standard cell preparations from the same samples as used for Western blot were stained for cyclin B1 with GNS1-Alexa Fluor 647 (A647) and DNA (DAPI). Arrows indicate the mean cyclin B1 fluorescence. Background fluorescence was subtracted and data were plotted as a hyperlog transformation which allows plotting of negative and zero values on a linear lower section (−10 to 10) and higher values (>10) logarithmically [22].

To determine the number of molecules of cyclin B1 in the standard cell preparations, the preparations and purified, recombinant cyclin B1 were electrophoresed on the same gel and blotted on the same paper. The range of the purified cyclin B1 and the number of cells per lane were adjusted so that each cell band was within the range of the purified standards. Each lane was adjusted to equal protein content by adding trypsin inhibitor. A representative Western blot of recombinant cyclin B1 with some standard cell preparations is shown in Figure 5A. To quantify Tag, we had adjusted the standard and the amount of cell lysate to give a narrow range of intensities to accommodate the narrow linear range of X-ray film. For cyclin B1 we used direct chemiluminescence detection with the FluorS MultiImager, which is linear over 3 orders of magnitude, so a wide range standard curve could be generated. The relationship between band intensity and the amount of cyclin B1 applied to each lane was determined by linear regression. The band intensity, the number of cells per lane, the regression slope and intercept, and the molecular weight of cyclin B1 were then used to calculate the average number of cyclin B1 molecules per cell for each standard cell lysate (Table 1). From 3 to 5 determinations were made for each cell line. The average coefficient of variation (CV) for all of the cell lines in the Western blot method was 24±7.5% (N = 9). Throughout this procedure a single lysate was used for each cell line. Therefore, this CV is an assessment of technical variation.

**Table 1:** Standard Curve.

	Western			Cytometer		
Cell line	Molecules/cell	SE	N	Fluorescence	SE	N
K652 100% NDZ	2455442	420471	5	104	13.1	2
PC3 100% NDZ	1367914	237408	4	46	7.0	2
PC3 60% NDZ	717428	68438	4	33	5.4	2
PC3 30% NDZ	560111	64416	3	16	2.4	2
PC3 15% NDZ	361117	39964	3	11		1
22R	152219	8494	4	9	0.5	2

### Flow Cytometry of Standard Cells

**Figure 6:**
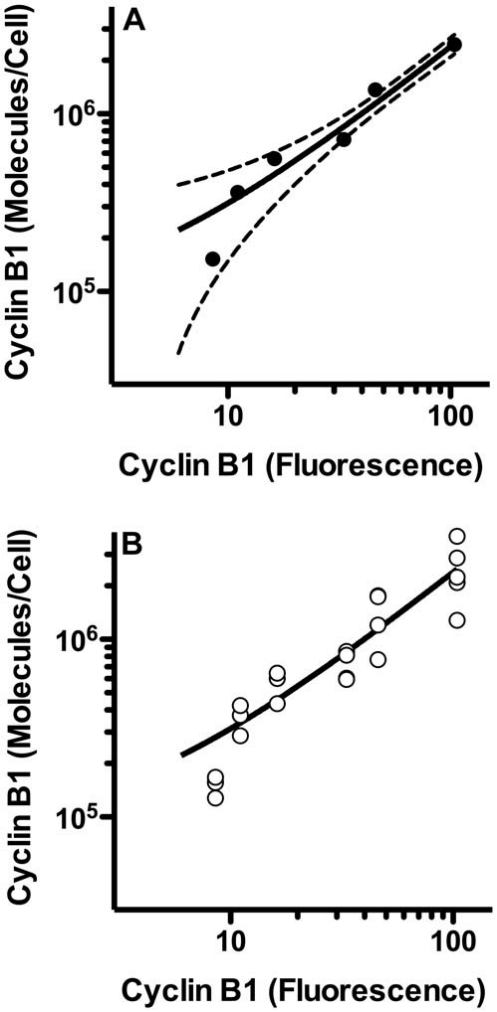
Correspondence between cyclin B1 measured by cytometry and by Western blotting. Average cyclin B1 levels were calculated from luminescence measurements of imaged Western blots then plotted versus mean fluorescence values (representative data shown in Figure 5). (A) Means and the 95% confidence interval are plotted. Cytometry was performed in duplicate. Western blots varied from 3 to 6 replicates. Individual values are plotted in (B).

Representative bivariate plots of the flow cytometric data corresponding to the same cell populations used for the lysates are shown in Figure 5B. Replicate samples were evaluated. G1 cells are essentially negative for cyclin B1 during the period of APC/C^Cdh1^ activity (from anaphase – through some point in G1). These cells were used for subtraction of non-specific binding of antibody and autofluorescence (see Materials & Methods). The range of average specific fluorescence values for the standard cell preparations was ∼12 fold (highest/lowest), while the range of average cyclin B1 levels by Western blots was ∼16 fold for the same samples. In similar comparisons of Tag, cytometry produced ∼4 fold range while Western blotting produced a 25 fold range [6]. This equates to a 1.3 fold difference in range ratio here and an ∼6 fold range ratio previously (Western range/cytometry range). We interpret this to indicate that the cytometry here was improved over the previous study. The Tag study differed with (1) a higher affinity antibody, (2) secondary immunostaining, (3) fluorescein as the fluorophore (compared to Alexa Fluor 647), and (4) X-ray film (compared to CCD camera). Of these, it seems likely that use of the brighter Alexa Fluor antibody that provides more photons in a spectral region with less autofluorescence, and perhaps less non-specific antibody staining (the antibody is used at a significantly lower staining level and the fluor does not carry a net charge). Analysis of the same standard cell preparations for cyclin B1 in this study using primary or secondary immunostaining with fluorescein isothiocyanate conjugated antibodies displayed only an approximately 2 fold range ratio, supporting this idea. The correlation between cytometry in relative fluorescence units and Western blotting (converted to molecules per cell) for the standard cell preparations is shown in Figure 6).

### Validation of Method with Test Cell Lines

**Figure 7:**
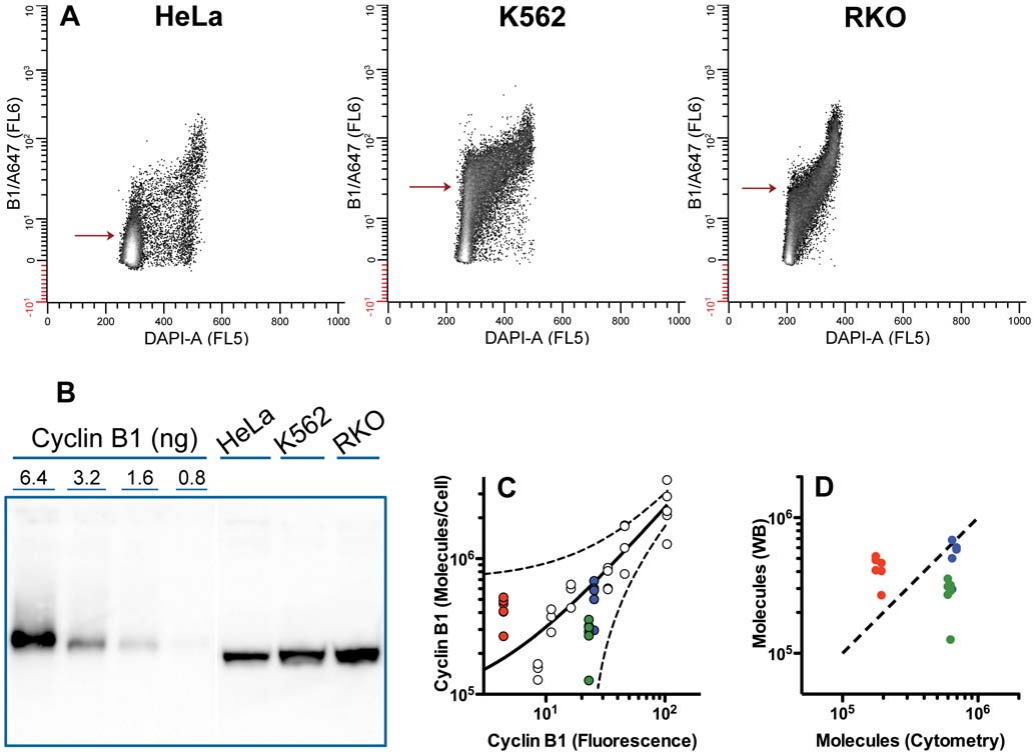
Test cell lines. Samples of HeLa, K562, and RKO were fixed with methanol then stained for cyclin B1 with GNS1-AlexaFluor 647 (A647) and DNA (DAPI). Part of the sample was also lysed and subject to Western blot analysis in the same manner as the standard lysates described in Figure 5. (A) cytometry data are displayed as in Figure 5. Arrows indicate the mean cyclin B1 related fluorescence. (B) Western blot prepared as in Figure 5. The lanes for the test cell lines were not loaded with equal cell numbers; loading was adjusted to obtain bands with intensities within the range of the cyclin B1 standards. Protein concentration was equalized with trypsin inhibitor. (C) co-plot of the standard curve and the test samples using the measured values for cyclin B1 molecules obtained by Western analysis for the test samples. HeLa = red circles; K562 = blue circles; RKO = green circles. The 95% prediction band for the standard curve is plotted (dashed lines). (D) Molecules were determined by Western blotting (Y axis) and by cytometry using the standard curve (X axis). The dashed line is a one-to-one correlation. Color coding is as in C.

To test the idea for using standard cells for cytometric quantification of unknown samples, we prepared MeOH-fixed samples of Hela, RKO, and K562 cells and quantified cyclin B1 by cytometry using the lyophilized standard preparations. The test samples were prepared and assayed in duplicate at the same time as the data for Figure 6. Representative bivariate plots of cyclin B1 versus DNA are shown in Figure 7A. We also prepared and assayed lysates of these same cell preparations. Six Western blots were performed. A representative blot is shown in Figure 7B. A plot of fluorescence versus molecules per cell (determined by blotting) is shown in Figure 7C (colored circles) plotted with the individual values for the standard curve (white circles). A plot of molecules per cell determined by cytometry and by Western blots is shown in Figure 7D. Determination of molecules by cytometry and the standard curve agreed with Western blotting for K562 (cytometry = 1.2 X Western). However, for RKO, the cytometry/standard curve method produced a mean that was 2.2 X that of the Western blots. For HeLa cells, cytometry = 0.4 X Western. These differences are acceptable when compared to other efforts to measure cyclin B1 (see below), and the values fall within the 95% prediction interval for the standard curve (Figure 7C). However, the error seem biased for the RKO and HeLa determinations. Rather than being distributed evenly, all determinations except one fall on one side or the other of the calculated correlation. If this bias is real, we do not know the source, however, for HeLa cells we have a speculative explanation. The HeLa sample was very confluent with 92% of the cells in G1. This means that a majority of the cells express levels of cyclin B1 that are below the threshold of detection by cytometry (this is estimated to be below 3–5% of the maximum expression of cyclin B1 for HeLa cells [19]). In this case, the higher sensitivity of Western blotting could result in measuring the population average of cyclin B1, whereas cytometry will register a large fraction of cells as zero (on average). If this is substantiated, this approach is limited when populations have high fractions of cells that express biomarker levels near the threshold of detection by cytometry. Also, if substantiated, it may be possible to derive a correction for this, at least for cyclin B1 and similar epitopes, by determining the relationship between degree of underestimate and cell density.

### Cell Cycle-related Expression of Cyclin B1

**Figure 8:**
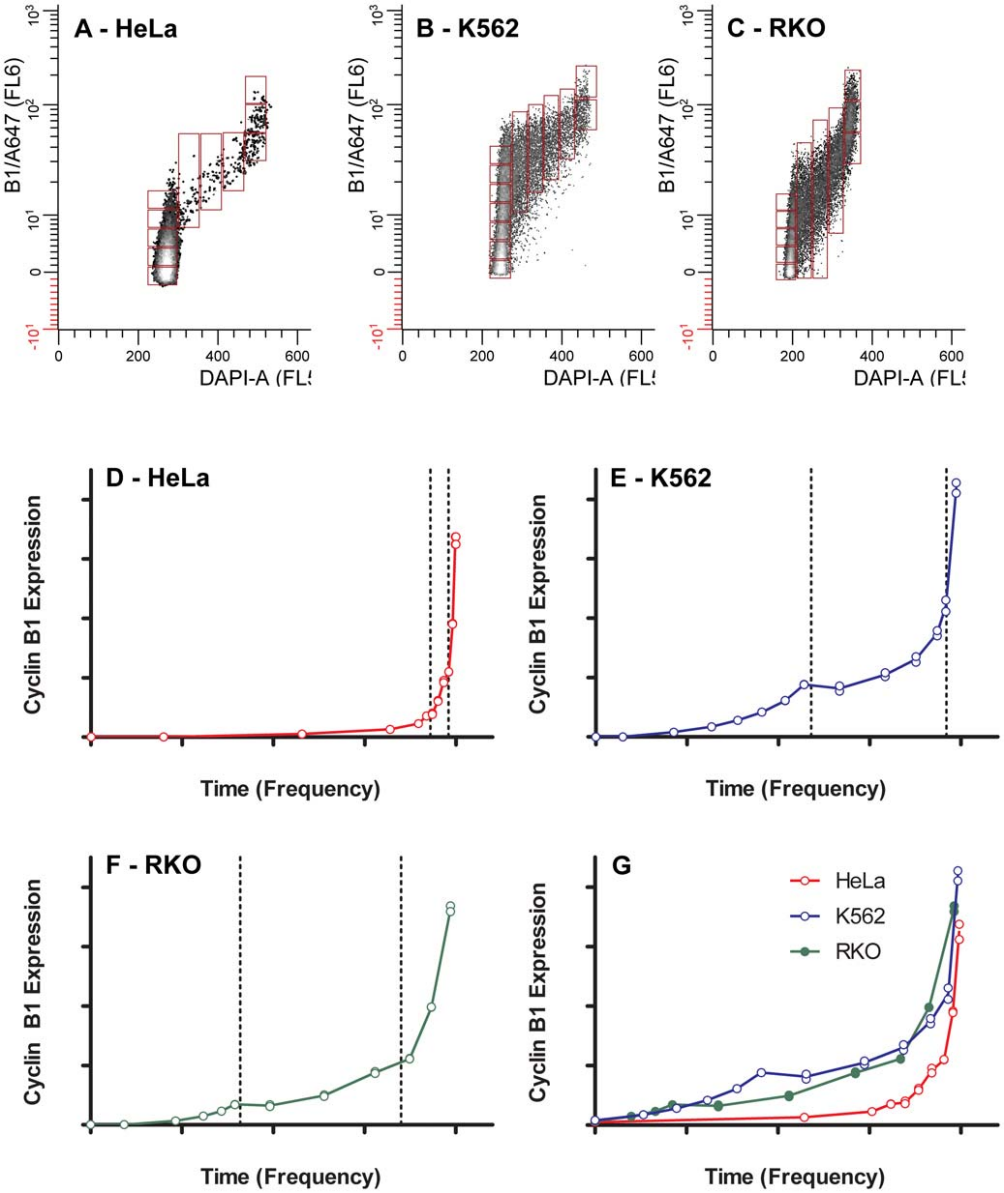
Cell cycle expression of cyclin B1. The cytometric data for HeLa, K562, and RKO cells (Figure 7) were gated to exclude endoreduplicated or binucleate cycling cells (4C 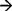 8C); mitotic cells after metaphase, and outliers. The purpose was to expose only the cells distributed about the two dimensional center as a function of DNA content. These data represent the most common cycling cell in the population. Arbitrary regions were set, moving through the data from early G1 through late G2+M (A–C). The mean cyclin B1 levels from each region are plotted as a function of the cell frequency in the region (D–F). The frequency data were renormalized from the first non-zero cyclin B1 point and plotted to compare the committed part of the cell cycle (cyclin B1 is detectable) for each cell line (G). In A-G, the X axis scale is 0–1, and the Y axis scale is 0–4×10^6^.

Cyclin B1 increases non-linearly throughout the cell cycle [10]. This dynamic range is not obvious with Western blotting of unsynchronized cells but is revealed by cytometry when addition of DAPI is used to measure correlated DNA content and provide measurements of the mean or median fluorescence for the G1, S, G2+M phases of the cell cycle. Using the relationship described by the data of Figure 6 to convert fluorescence to molecules, we determined the expression of cyclin B1 in molecules per cell as a function of progression through the cell cycle for the three test cell line samples. These data were trimmed to include only the simple 2C cycle of G1 through metaphase. Figure 8A shows the arbitrary regions used to determine the local cyclin B1 average and cell frequency at that average. The cyclin B1 region specific fluorescence was converted to molecules and plotted as a function of cell frequency, which is proportional to time (Figure 8E–G). Because cyclin B1 increases dramatically in the G2 phase of the cell cycle, we anticipated that conversion to molecules as shown in Figure 8 might be problematic if the region specific fluorescence values were outside the range of the standard curve. However, since the NDZ-treated cell lines extended the range of Western blot measurements at the high end to supra normal levels, we did not encounter this uncertainty.

To show the similarity of expression in greater detail, Figure 8G shows the cell cycle related expression for all three samples starting at the first significant value greater than 2% of max. We take this to demark a position after the loss of Cdh1 activity and synthesis of the mitotic B cyclins (we cannot detect cyclin A2 at this time in any cell line that we have examined). This should also be within the committed part of G1, although this is speculative. It may be useful to note that despite the fact that these are three highly cultured human tumor cell lines of different lineages (myeloid, colon, cervical) and even though the HeLa cells were growing very slowly as a population, the end point cyclin B1 levels are remarkably close. This suggests tight quantitative regulation in an absolute sense at the point at which the cyclin B1/Cdk1 complex becomes active.

### Other Approaches of Cyclin B1 Quantification

Data from other quantifications of cyclin B1 are listed in Table 2. Each group used different methods, but ultimately relied on Western blots for final quantification. There is a very wide range of levels reported and some uncertainty about the exact size of the cells within any one study, but if we either use the reported concentrations or use cell size values that are reasonable, the resulting cyclin B1 concentrations fall into groups with a difference of 3 orders of magnitude between them. The yeast data [14] seem to fall in an intermediate position, but these may be underestimates. This group tagged all the proteins of interest with protein A in order to quantify them against purified protein A and used protein A antibody in Western blot detection. When this method was checked with some untagged or myc-tagged proteins, the protein A values were found to be lower in 3 out of 4 instances. This suggests that the protein A tag may be reducing net expression levels for some of the proteins.

**Table 2:** Comparison of Cyclin B1 Quantification.

Cells	Phase/Stage	Molecules/cell	Diam. (u)	Conc.(uM)	Ref.
HeLa	G2+M	a2×10^6^	b15	1.8	This study
HeLa	Prometaphase	3×10^6^	15	2.9	[4]
HeLa	G2	c4×10^3^	15	0.004	[11]
Mouse oocytes	Incompetent	10^7^	d55	0.2	[12]
	Competent	10^8^	d79	0.6	
Xenopus oocytes	stage VI	4×10^8^	e1280	0.0006	[13]
Yeast	all (Clb2)^f^	1×10^3^	g4.3	0.04	[14]
	all (B cyclins)	4×10^3^	g4.3	0.15	

a actual value = 1.6×10^6^.

b Used the value of Xu et al. [4]

c Calculated from the published values of pmole cyclin B1/g extract and 2×10^3^ cells/ug lysate.

d Measured by Kanatsu-Shinohara et al. [12].

e Estimated from molecules and concentration given by Kobayashi et al. and diameter given by Chang et al. [23].

f cyclin type in parentheses.

g Measured by Jorgensen et al. [24].

All of these methods relied on Western blots for the final quantification step, but used different variations. Some groups did not control for total protein level. Some used ratios instead of standard curves. But these differences seem minor compared to the large gap in reported cyclin B1 concentrations. The two groups with the lowest expression [11], [13] used radioactivity to quantify their standard, which was generated in a reticulocyte lysate. We have noted endogenous B cyclin in reticulocyte lysates, but both studies checked the values generated by reticulocyte lysates against recombinant protein expressed in *E. coli*, so this seems an unlikely source of error.

Despite careful reading of these papers, we do not see anything technical that would account for the thousand-fold differences in cyclin B1 values. For the study of Kobayashi et al., the variation could be biology. If so, then Xenopus oocytes operate on much less cyclin B1 compared to mammalian somatic cell lines. Since HeLa cells were used in this study and that of Arooz et al. and Xu et al. [4], the differences are particularly troubling. We would expect that Xu et al. and our study would measure slightly higher levels for G2+M cells since both studies employ cell based measurements with enhanced ability to measure G2 and M, but this does not explain the difference between these studies and that of Arooz et al. We have preliminary data for an independent method similar to that of Xu et al. We have used the fluorescence of a commercial source of purified EGFP to quantify the expression of EGFP-cyclin B1 expressing HeLa cells, and then used Western blots to quantify endogenous cyclin B1 relative to EGFP-cyclin B1. The preliminary results support the higher values of endogenous cyclin B1 that we have reported herein.

## Materials and Methods

### Purification of recombinant Cyclin B1

Baculovirus infected insect cells expressing recombinant human cyclin B1 was a generous gift from Susan Wormsley (PharMingen, San Diego, CA). To purify cyclin B1, 2.5×10^8^ cells were suspended in 7 ml lysis buffer [50 mM Tris HCl, pH 7.0, 150 mM NaCl, 0.1% Na Azide, 2 mM phenylmethylsufonyl fluoride (PMSF) and 10 µl/ml protease inhibitor cocktail (Sigma P8340)]. An equal volume of lysis buffer with 2% Nonidet P-40 (NP-40) was added, and the lysate was stirred for 1 h at 4°C. Insoluble material was removed by centrifugation at 15,000 g for 15 min at 4°C; 1 ml aliquots of supernatant were frozen in a dry ice/EtOH bath and stored at −80°C.

Anti-cyclin B1 monoclonal antibody (GNS1, PharMingen) was conjugated to Affi-Gel Hz (BioRad, Hercules, CA) as directed. Four mg IgG was oxidized and coupled to 4 ml of Affi-Gel Hz. Absorbance at 280 nm was used to quantify coupling efficiency (44%).

Lysate was added to the affinity column and the column was washed using lysis buffer with 0.5% NP-40 (solution A) then with solution A with 0.5 M NaCl until A_280_ returned to base line. Cyclin B1 was eluted with 1M sodium propionate, 150 mM NaCl, 10% dioxane, and 0.5% NP40 at pH 5.0 (solution E). A shallow gradient to 50% solution E followed by a steep gradient to 100% solution E was used to collect fractions. Samples were frozen in dry ice/ethanol and stored at −80°C. Fractions were monitored by Western blotting. Cyclin B1 fractions were pooled and concentrated in 30K MW MSI Ultrafuge (Westboro, MA) centrifuge filters. The concentrate was subjected to anion exchange chromatography as follows. Pooled fractions were re-equilibrated in solution A without NP-40 (EconoPac 10 DG, BioRad) then concentrated in MSI Ultrafuge or Millipore (Bedford, MA) Ultrafree-4 centrifuge filters and mixed with a Macro-Prep High Q Support (BioRad) anion exchange resin in solution A. After centrifugation the preparations were concentrated again to less than 1 ml.

### Cell Lines and Culture

PC3, DU145, RKO, HeLa, and 22Rv1 were grown in Dulbecco's modified Eagle medium (DMEM) with 5% FBS and 5% calf serum (Sigma, St. Louis, MO). K562 was grown in RPMI 1640 (Gibco, Grand Island, NY) with 10% FBS. All cultures were maintained at 37°C in 5% CO_2_.

### Cell line lysates

Adherent cells harvested with trypsin-EDTA or nonadherent cells were counted with a Coulter Counter (Coulter, Hialeah, FL), and then washed in PBS. Lysis buffer was added to give a final cell concentration of between 5×10^6^/ml and 5×10^7^/ml, depending on the cyclin B1 level in the cell line. Cells were lysed with SDS lysis buffer (1% sodium dodecyl sulfate (SDS), 2% NP-40, 1% Na deoxycholate, 137 mM NaCl, 20 mM Tris HCl, pH 7.5) and DNA was sheared by syringing with a 26-gauge needle. SDS concentration was varied to 5 or 23%. Other extraction buffers used were RIPA (1% Na deoxycholate, 0.1% SDS, 1% Triton X-100, 1 mM EDTA, 150 mM NaCl, 10 mM Tris HCl, pH 7.5), TX-100 (0.75% Triton X-100, 0.5 M NaCl, 1 mM EDTA, 10% glycerol, 20 mM Tris HCl, pH 8) and NP-40 (0.5% NP-40, 5 mM MgCl_2_, 120 mM NaCl, 5 mM Tris HCl, pH 7.5). All lysis buffers contained 2 mM PMSF and 10 µl/ml protease inhibitor cocktail (Sigma). Extracts were stored at −20°C.

Protein content was measured by protein precipitation with UPPA-1 and UPPA-2 (Geno Tech, St. Louis, MO) according to the manufacturer's directions; re-solubilization (0.1% SDS, 1% deoxycholate, 0.5M NaOH), followed by the Lowry method [20]. BSA was used for the standard curve.

### Quantitative Western Blotting

Western blotting was done as described previously [6]. The primary antibody was 1.5 µg α-cyclin B1 clone GNS1 (PharMingen) and the secondary was 5 µl alkaline phosphatase conjugated goat-α-mouse IgG (Promega, Madison, WI) in 15 ml blocking buffer. The substrate was CDP-star (Tropix, Bedford, MA). Blots were imaged with a Flour-S MultiImager (BioRad) and images analyzed with Quantity One software (BioRad).

### Cell Fixation and Lyophilization

Single cell suspensions were fixed in 0.5% formaldehyde for 10 min at 37°C followed by 90% methanol at −20°C. Fixed cells were lyophilized as individual samples in 1.5 ml centrifuge tubes on a Labconco Freezone 12 Liter Freeze Dry System, model 77540 (Kansas City, MO) using histidine lyophilization buffer (5 mM histidine, 0.1% Tween 20, 2 mM sucrose). Lyophilized samples were stored in desiccators at −80°C. Samples were rehydrated with distilled water equal to the amount of lyophilization buffer used.

### Immunofluorescence measurement

Samples were stained for cyclin B1 and DNA as previously described [21]. Briefly, 5×10^5^ cells were incubated with 0.25 µg GNS1, followed by washing, then incubated with 1.25 ug FITC-conjugated goat anti-mouse IgG (1.25 µg). RNA was digested with RNase and then propidium di-iodide was added at a final concentration of 50 µg/ml. Cells were analyzed on a Coulter EPICS XL- MCL Cytometer (Coulter Electronics, Miami, FL). In some experiments, cells were stained with GNS1 conjugated to Alexa Fluor 647 and phycoerythrin conjugated cyclin A2 antibody (gift from Vince Shankey, Beckman Coulter, Miami, FL); DNA was stained with DAPI, and cells were analyzed on a BD LSR I Cytometer (BD Biosciences, San Jose, CA).

### Data analysis

All data primary analysis was performed offline with WinList 6.0 (Verity Software House, Topsham, ME), and non-linear regression was performed with GraphPad 5.0, GraphPad Software, San Diego California USA).

Background Subtraction: To reduce background to near an average of zero, we subtracted median fluorescence as a function of cyclin B1 expression for G1 cells. Since cyclin B1 is not expressed for much of G1, this provided a near perfect control for background binding of the GNS1 antibody. This was accomplished in WinList (Verity Software House, Topsham, ME) using compensation algorithms.

### Calculation of molecules per cell

Optical densities (OD) from CCD exposure to Western blot chemiluminescence from known numbers of cyclin B1-expressing cells and from a serial dilution of purified recombinant cyclin B1 were used to determine the average molecules of cyclin B1 per cell. Cytometry was used to obtain the average cyclin B1-related fluorescence per cell for the same cell lines (duplicate cultures) used in the Western blots. Linear regression was used to relate OD to molecules and fluorescence to molecules for the standard and test cell lines [6].

### Calculation of cell cycle expression of cyclin B1

After background subtraction, gates on cyclin A2 versus light scatter, DNA content versus light scatter, and cyclin B1 versus DNA content were used to isolate only the 2C cycling component from G1 through metaphase. Contiguous arbitrary regions were set on cyclin B1 versus DNA content bivariate histograms from early G1 through G2+M. Mean cyclin B1 levels per region were plotted versus a cumulative function (1) of the cell frequency per region.
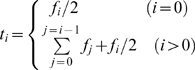
where *f* =  the frequency of cells within a region; *i* =  the region index beginning at 0 and progressively moving through the cell cycle.
